# Phenytoin Pharmacokinetics During Venoarterial Extracorporeal Membrane Oxygenation and Plasma Exchange

**DOI:** 10.7759/cureus.17120

**Published:** 2021-08-12

**Authors:** Joseph C Osborne, Caitlin S Brown, Nathan D Peffley, Erica D Wittwer, Suraj M Yalamuri

**Affiliations:** 1 Pharmacy/Clinical Pharmacist, Mayo Clinic, Rochester, USA; 2 Pharmacy and Emergency Medicine, Mayo Clinic, Rochester, USA; 3 Anesthesiology, Mayo Clinic, Rochester, USA

**Keywords:** plasma exchange, anti-phospholipid syndrome, phenytoin, extracorporeal membrane oxygenation, catastrophic antiphospholipid syndrome

## Abstract

Currently, there is minimal guidance to antiepileptic dose adjustment for a patient requiring either venoarterial (VA) extracorporeal membrane oxygenation (ECMO) or plasma exchange (PLEX) therapy, and to our knowledge, there are rare guidances for a patient requiring both. Given the dangers with non-therapeutic concentrations of phenytoin, it is critical for the intensive care unit (ICU) practitioner to understand how the pharmacokinetic parameters of phenytoin change in critically ill patients requiring extracorporeal support.

This case study presents a 41-year-old female transferred to the cardiovascular ICU requiring VA ECMO and PLEX for the treatment of systemic lupus erythematosus (SLE)-induced catastrophic antiphospholipid syndrome (CAPS). Free phenytoin concentrations were measured to assess the removal of phenytoin. There was no significant decrease in the free phenytoin concentrations post-PLEX and while on ECMO.

Free phenytoin concentrations are not influenced in the setting of PLEX and while on ECMO.

## Introduction

This case will describe phenytoin dosing and concentrations in a patient with a history of epilepsy on venoarterial (VA) extracorporeal membrane oxygenation (ECMO) in the setting of plasma exchange (PLEX) secondary to systemic lupus erythematosus (SLE)-induced catastrophic antiphospholipid syndrome (CAPS) via monitoring of free phenytoin concentrations. With phenytoin being a highly protein-bound medication, both ECMO and PLEX run the risk of removing bound phenytoin. In addition, phenytoin has a low volume of distribution, leading to a higher presence of the free drug in plasma, thus increasing removal of the drug during a PLEX procedure. Considering these pharmacokinetic factors alongside that the patient required sedation during much of her intensive care unit (ICU) stay, there was a concern for both epileptic activity unrecognizable via physical symptoms and potential phenytoin toxicity, prompting evaluation of therapeutic effectiveness of phenytoin via free concentrations. The patient provided written HIPAA (Health Insurance Portability and Accountability Act) authorization to publish this case report.

## Case presentation

A 41-year-old 125-kg female of an undisclosed race was emergently transferred to the cardiovascular surgery ICU after VA ECMO cannulation at an outside hospital due to cardiogenic shock in the setting of bilateral pulmonary emboli, hemodynamic instability secondary to atrial fibrillation with a rapid ventricular response, and a large left atrial thrombus. Fibrinolytics were administered at an outside facility resulting in epistaxis and hemothorax. After transfer, hemostasis was obtained, then heparin anticoagulation for ECMO and the known emboli was initiated, and diagnostic workup began from the cause of massive thrombosis.

Pertinent past medical history included a previous traumatic brain injury without residual deficit and chronic seizure disorder controlled on oral levetiracetam (750 mg twice daily) and extended-release phenytoin (200 mg twice daily). Upon arrival, the patient started 200 mg phenytoin equivalents of fosphenytoin via intravenous (IV) route twice daily administered over 15 minutes and 750 mg oral solution of levetiracetam twice daily via gastric tube. Antiphospholipid IgM was found to be positive on day four of stay, resulting in suspicion and confirmation of SLE-induced CAPS. Profound cardiogenic shock was managed with VA ECMO, whereas definitive CAPS treatment could be administered for the treatment of the primary disease. Aggressive treatment with high-dose methylprednisolone, IV immunoglobulin therapy, and PLEX were initiated for SLE-induced CAPS, with PLEX therapy occurring between days seven and 13 for a total of seven treatments. Repletion fluid during PLEX consisted only of fresh frozen plasma; albumin was not used. The patient did not require hemodialysis.

No epileptic activity was documented throughout the patient’s course in the hospital. Total and free phenytoin concentrations were monitored throughout the patient’s course of PLEX and VA ECMO to ensure therapeutic efficacy and safety. Phenytoin concentrations, along with serum albumin, are shown in Table 1. Free phenytoin concentrations remained therapeutic throughout PLEX treatment. Shock resolution occurred after completion of PLEX therapy, and VA ECMO decannulation took place on day 20 of the patient stay. The ECMO circuit and thrombus were managed with heparin anticoagulation utilizing heparin anti-Xa monitoring due to interference between antiphospholipid syndrome (APS) and partial thromboplastin time (aPTT). A target anti-Xa goal of 0.3 to 0.5 IU/ml was utilized. The patient was free of epileptic activity throughout her hospitalization and was discharged to a rehabilitation facility in stable condition.

**Table 1:** Phenytoin Concentrations PLEX, Plasma exchange. The therapeutic range of free phenytoin: 1-2 mcg/mL. The therapeutic range of total phenytoin: 10-20 mcg/mL.

Timeline of Lab Draw (Inpatient Day)	Timing of Draw Post-PLEX (min)	Free Phenytoin Concentration (mcg/mL)	Total Phenytoin Concentration (mcg/mL)	Serum Albumin (g/dL)
First Draw Pre-PLEX (7)	N/A	2.4	7.9	2
First Draw Post-PLEX (7)	3	1.6	8.6	
Second Draw Pre-PLEX (8)	N/A	1.6	8.8	2.5
Second Draw Post-PLEX (8)	455	1.7	9.4	
Third Draw Pre-PLEX (13)	N/A	1.4	8.2	2.9
Third Draw Post-PLEX (17)	6,516	1.4	8.2	2.6
Routine Draw (23)	N/A	1.3	5.3	2.1

## Discussion

In this case, the patient required both ECMO and PLEX therapies that can augment the clearance of highly protein-bound medications, such as phenytoin. Lowered seizure threshold secondary to these necessary therapeutic interventions was of concern. Phenytoin is complicated to dose due to non-linear pharmacokinetics. A thoughtful approach to dosing phenytoin must be pursued in the setting of narrow therapeutic index and risk of phenytoin toxicity [1,2]. Therapeutic ranges for phenytoin consist of 10-20 mcg/mL for total phenytoin and 1-2 mcg/mL for free phenytoin serum concentrations [3]. Critically ill patients have disruptions in homeostatic metabolism influenced by pro-inflammatory cytokines and experience fluctuating albumin levels, consequently altering free phenytoin levels [1-3]. Given the frequency of hypoalbuminemia and renal dysfunction in critically ill patients, monitoring of phenytoin toxicity is critical to consider [1-3]. An accompanying complexity of this case included the use of PLEX for the treatment of SLE-induced CAPS, which has been reported in influencing drug levels [4,5]. Briefly, PLEX consists of centrifugation of whole blood followed by separation into plasma and various cellular components, removal of plasma, and subsequent replacement with fresh frozen plasma and/or albumin [5]. This method of selective filtration allows for specific targeting of pathogenic blood components including antibodies [5]. Given the nature of PLEX, pharmacologic therapies with a low volume of distribution experience an amplified likelihood of removal due to their increased presence in plasma [4,5]. Phenytoin has a low volume of distribution, ranging from 0.52 to 0.78 L/kg, thus imposing theoretical risk for sub-therapeutic concentration following PLEX [6,7].

Protein binding is another pharmacokinetic factor that must be taken into consideration with PLEX and ECMO sequestration of medications. Phenytoin retains a high affinity for adsorption onto protein, with 87.9%-91.9% of the total dose being bound [8]. Therapeutic agents that bind to proteins contained in blood, such as albumin, are more readily removed from plasma, resulting in undesirable concentrations following PLEX [4,5]. ECMO removal of medications is similar in this regard as lipophilic and/or protein-bound medications can become trapped within the oxygenator membrane [9]. While phenytoin is hydrophilic, it is highly protein-bound and thus at an additional risk of removal via ECMO [9]. Given the unpredictability of PLEX and ECMO on the equilibrium between free and bound phenytoin concentrations, a method to monitor phenytoin levels for efficacy and safety is needed [1].

PLEX effect on phenytoin-free and total concentration in the setting of ECMO is known through a small number of case studies. One such 1982 report described the PLEX effect on phenytoin in a patient afflicted with thrombotic thrombocytopenic purpura, in which a constant fraction (10%) of total body phenytoin was removed with every two passes of PLEX [10]. The consequence of this removal was a 160% increase from the calculated phenytoin dose, and the authors thus concluded PLEX has a clinically significant impact on phenytoin therapy [10]. Moreover, a 1985 case study measured total and free phenytoin levels in two patients receiving PLEX in the setting of myasthenia gravis [11]. Both patients were not found to have statistically significantly different total or free phenytoin concentrations when comparing draws two hours post-PLEX and one day post-PLEX [11]. The authors conclude that additional phenytoin dosing was not required and that free or total phenytoin concentration was not being significantly impacted post-PLEX [11]. A 1986 report highlighted that rapid equilibration between bound and free phenytoin occurs, thus making dose adjustments of phenytoin therapeutically unnecessary [12]. Further, a 2020 article reported that dose adjustment of fosphenytoin is not necessary with PLEX with the exception of patients undergoing plasma exchange for greater than two days [13]. Literature in the adult patient population to date regarding ECMO effect on phenytoin pharmacokinetics is scarce, yet one 2019 retrospective chart review examined two patients using phenytoin and requiring ECMO and recommended monitoring free serum phenytoin, given labile total phenytoin concentration [14].

To navigate this complex drug elimination scenario, we elected to measure total and free phenytoin concentration pre- and post-PLEX. Phenytoin total and free levels were collected from serum and measured alongside serum albumin to assess the relationship between PLEX and quantitative free phenytoin concentration. Bound levels of phenytoin were found to be consistently below the target range of 10-20 mcg/mL, but free phenytoin concentration remained steady following PLEX within the goal range of 1-2 mcg/mL. The net balance of all these factors showed that while the bound drug was removed by PLEX along with proteins (albumin), the free drug was able to remain therapeutically unaffected. This stable therapeutic level and unchanged dosing of phenytoin indicate that PLEX therapy with ECMO had little impact on free phenytoin concentration in our patient.

## Conclusions

The guidance this case study provides for the ICU practitioner for monitoring phenytoin in a patient on VA ECMO in the setting of PLEX is impactful. Previous studies clash regarding the influence PLEX or ECMO has on phenytoin concentration, let alone the consequence of both PLEX and ECMO. We found that the combination of PLEX and VA ECMO lowered total phenytoin concentration below the therapeutic level but did not lower free phenytoin below the therapeutic level. Additionally, we revealed that measuring pre- and post-PLEX phenytoin total and free levels was sufficient in monitoring the therapeutic concentration while the patient was sedated. This information is useful to ICU providers with patients requiring multifaceted mediums of care such as PLEX and VA ECMO.
